# Decision Making about Localized Esophageal Cancer Treatment: An Observational Study on Variation in Clinicians’ Communication Behavior

**DOI:** 10.1177/23814683251349473

**Published:** 2025-06-30

**Authors:** L. F. van de Water, G. C. Scholten, I. Henselmans, J. Heisterkamp, P. M. Jeene, F. F. B. M. Heesakkers, K. J. Neelis, B. R. Klarenbeek, M. I. van Berge Henegouwen, J. W. van den Berg, J. Buijsen, E. D. Geijsen, H. W. M. van Laarhoven, E. M. A. Smets

**Affiliations:** Department of Medical Oncology, Amsterdam UMC location University of Amsterdam, Amsterdam, the Netherlands; Department of Medical Psychology, Amsterdam UMC location University of Amsterdam, Amsterdam, the Netherlands; Amsterdam Public Health, Quality of Care, Amsterdam, the Netherlands; Cancer Center Amsterdam, Cancer Treatment and Quality of Life, Amsterdam, the Netherlands; Department of Medical Oncology, Amsterdam UMC location University of Amsterdam, Amsterdam, the Netherlands; Department of Medical Psychology, Amsterdam UMC location University of Amsterdam, Amsterdam, the Netherlands; Department of Medical Psychology, Amsterdam UMC location University of Amsterdam, Amsterdam, the Netherlands; Amsterdam Public Health, Quality of Care, Amsterdam, the Netherlands; Cancer Center Amsterdam, Cancer Treatment and Quality of Life, Amsterdam, the Netherlands; Department of Surgery, Elisabeth-TweeSteden Ziekenhuis, Tilburg, The Netherlands; Department of Radiation Oncology, Radiotherapiegroep, Deventer, the Netherlands; Department of Surgery, Department of Intensive Care Medicine, Catharina Hospital, Eindhoven, The Netherlands; Department of Radiation Oncology, Leiden University Medical Center, Leiden, the Netherlands; Department of Surgery, Radboud University Medical Centre, Nijmegen, Netherlands; Cancer Center Amsterdam, Cancer Treatment and Quality of Life, Amsterdam, the Netherlands; Department of Surgery, Amsterdam UMC location University of Amsterdam, Amsterdam, the Netherlands; Department of Surgery, University Medical Center Utrecht, Utrecht, The Netherlands; Department of Medical Psychology, Amsterdam UMC location University of Amsterdam, Amsterdam, the Netherlands; Department of Radiation Oncology (MAASTRO), GROW School for Oncology and Developmental Biology, Maastricht University Medical Centre, Maastricht, the Netherlands; Cancer Center Amsterdam, Cancer Treatment and Quality of Life, Amsterdam, the Netherlands; Department of Radiation Oncology, Amsterdam UMC location University of Amsterdam, Amsterdam, the Netherlands; Department of Medical Oncology, Amsterdam UMC location University of Amsterdam, Amsterdam, the Netherlands; Cancer Center Amsterdam, Cancer Treatment and Quality of Life, Amsterdam, the Netherlands; Department of Medical Psychology, Amsterdam UMC location University of Amsterdam, Amsterdam, the Netherlands; Amsterdam Public Health, Quality of Care, Amsterdam, the Netherlands; Cancer Center Amsterdam, Cancer Treatment and Quality of Life, Amsterdam, the Netherlands

**Keywords:** decion making, patient-provider communication, cancer

## Abstract

**Background.** For localized esophageal cancer, more than 1 curative treatment option is available. As these different options are associated with substantially different treatment outcomes, decision making can be complex. Moreover, treatment decision making for a patient involves multiple health care providers (HCPs) from different disciplines over time, who might have their own role and perspective on the decision-making process. This study aims to describe how HCPs communicate during treatment decision consultations with patients with localized esophageal cancer. **Methods.** Audio recordings of 20 preintervention scripted standardized patient assessments (SPAs) from the SOURCE trial were used. Using 2 highly similar cases, acted by a simulated patient, considerably reduced variation at patient level. Audio recordings were content coded using open coding and rated on the degree of patient involvement in decision making using the OPTION-12. **Results.** Radiation and surgical HCPs discussed 1 to 4 different treatment options, from a total of 5 different options observed over all consultations. They discussed 0 to 11 different side effects and complications, from a total of 28. While some HCPs explicitly presented a choice, many used various implicit forms of suggesting a choice and either implicitly or explicitly marked their own preferences for treatment. Consultations showed a mean OPTION-12 score of 40.11 (range 0–100). **Conclusions.** This study shows extensive practice variation in how and to what extent standardized patients with localized esophageal cancer were involved in decision making and in the number and type of treatment options and pros and cons that were presented to them. **Implications.** The findings suggest a need for mutual alignment within oncologic HCPs treating patients with esophageal cancer.

**Highlights:**

- Practice variation was found in how and to what extent health care providers involved standardized patients with localized esophageal cancer in decision making.
- Health care providers varied in the number and type of treatment options and pros and cons they presented.
- These findings suggest a need for multidisciplinary alignment.

Esophageal cancer causes more than half a million deaths yearly worldwide.^1^ For patients with localized and resectable esophageal cancer, neoadjuvant chemoradiotherapy (nCRT) followed by surgery is standard of care.^2,3^ While this treatment option is associated with the highest chances of curation, 5-y survival rates do not exceed 50%.^4,5^ Although recently adjuvant nivolumab has shown improvement in disease-free survival in patients who did not achieve a complete pathological response after chemoradiotherapy, survival results are still limited.^6^ Importantly, esophagectomy is also associated with a risk of serious pulmonary or cardiac complications, anastomotic leakage, or, in rare cases, even mortality.^4^

Furthermore, esophagectomy comes with a considerable impact on the daily lives of patients, such as functional disorders of the gastric tube.^7^ Up to 80% of the patients experience long-term gastroesophageal reflux, causing them difficulties with sleeping in a horizontal position.^7–11^ Other frequently occurring symptoms, such as delayed or accelerated gastric-emptying and dumping syndrome, may cause patients to experience several complaints during or after food intake, such as regurgitation, pain, nausea, diarrhea, abdominal cramps, vertigo, and palpitations.^12–14^ It is usually only in the long term that patients win back their previous quality of life.^7,15^

Other options for treatment with curative intent exist, for instance, definitive chemoradiotherapy (dCRT), in which patients are treated with chemoradiotherapy to a higher dose, without having to undergo surgery.^16^ This treatment therefore lacks the symptoms due to functional disorders and the risk of complications linked to surgery. It is, however, associated with other morbidities and, importantly, substantially lower survival rates (approximately 15%).^16^

Since the pros and cons of the aforementioned treatment options might weigh differently for each patient, one could argue that it requires patients’ individual consideration in a shared decision-making (SDM) process.^17,18^ In most SDM models, the health care provider (HCP) presents a choice between multiple relevant options, informs about the benefits and burden of these options, and subsequently supports the patient in preference construction.^19,20^ SDM is particularly advocated for preference-sensitive decisions,^21,22^ for which there is no one “best” choice. However, different from many other treatment decisions in cancer care,^23,24^ the large difference in survival rates between the aforementioned options may result in a strong preference from a medical perspective.^19,25^ This might make the application of the SDM model less self-evident, as it would require HCPs to discuss 1 or more medically “inferior” options with patients. Therefore, communication about treatment decisions in the setting of localized esophageal cancer might be complex.

As treatment options often involve multiple treatment modalities (e.g., different combinations of chemotherapy, radiotherapy, and surgery), treatment decision making also involves multiple HCPs (e.g., medical oncologists, radiation oncologists, surgeons, nurse specialists, physician assistants), who may approach the decision-making process differently.

To date, little is known about the current practice of decision making in the setting of potentially curable esophageal cancer. The high disease-specific mortality rates, the substantial difference in outcomes between treatment options, and the complicated multidisciplinary setting, do, however, distinguish decision making for this disease from other oncologic settings studied thus far. The aim of this study is therefore to describe how HCPs communicate about treatment decisions with patients with localized esophageal cancer. More specifically, we want to investigate what information is provided to patients and in which way, how the way information is provided could affect patients’ perception of choice, and how patients are involved in decision making.

## Methods

### Design and Ethics

The presented analyses are part of a larger prospective multicenter effect study about treatment outcome communication in esophagogastric cancer: the SOURCE trial (NCT04232735). The SOURCE trial examines the effect of a prediction tool and communication training on physicians’ outcome communication by audio recording consultations of patients with esophageal or gastric cancer and performing standardized patient assessments (SPAs) before and after the intervention. Due to COVID-19, most of the SPAs were performed online (15/20) via video call (GoToMeeting). The SOURCE trial was declared exempt from review by the institutional medical ethics review boards of the study contractor (Amsterdam University Medical Centers, Medical Ethics Review Board AMC: W19_094) and approved by the local review boards of all study sites (Medical Ethics Review Board VUmc: 2019.501; Medical Ethics Review Board Utrecht, 20/173; Medical Ethics Review Board Leiden Den Haag Delft: N21.089). All methods were carried out in accordance with relevant guidelines and regulations.

For the current analyses, we used the audio recordings of the preintervention SPAs, which involved a case of an esophageal cancer patient opting for curative treatment, resulting in SPAs of surgical and radiation HCPs. Our intention was to allow for the inclusion of different disciplines within the multidisciplinary team involved in decision making (i.e., surgical and radiation HCPs), however, without the aim of comparing between them. As a result, we did not specifically sample different HCPs. During the SPAs, 2 highly comparable localized esophageal cases, of which only the background story and the type of comorbidity differed, were acted out by 2 simulated patients. Cases and actors were randomly assigned to the HCP. This design considerably reduced variation at the patient level and enabled us to focus on the decision-making behavior of the HCPs.^26,27^

### Participants

HCPs were eligible for the SOURCE trial if they regularly discussed treatment and treatment outcomes with patients and were used to giving this information to patients. HCPs were recruited from 8 different hospitals in the Netherlands, 4 of which were academic and 4 nonacademic hospitals. Informed consent was obtained from all participating HCPs. They were informed that the SOURCE study aimed to investigate the effect of an intervention on the way HCP inform patients about treatment outcomes, including survival, side effects and complications, and quality of life.

### SPA Cases

The standardized cases reflected a standard patient with localized esophageal cancer opting for curative treatment, who met the HCP to talk about treatment for the first time. The participating HCPs received a simulated medical file, containing the standard medical information noted down by the gastroenterologist.

According to the cases, the patient was 76 y old, had a mild comorbidity, chronic heart disease, or diabetes type 2, which was well-controlled (see Appendix 1 for a detailed description of the SPA cases). After the SPA, HCPs indicated the level of realism and comparability to clinical practice on a 1 to 10 Likert-type scale (1 = *very unrealistic/incomparable*, 10 = *very realistic/comparable*).

Two professional male actors with experience in performing in a SDM research context were trained based on a script containing patient background information and instructions.^27,28^ They were instructed to act in a standard way and to act rather passive and not overly emotional. The script included a set of standard questions and a few “if-then” rules (e.g., instructions to ask a certain question only in case the HCP presented a particular piece of information). They were instructed about their own treatment preferences, which was to prefer nCRT followed by surgery over other options. Both the patient scripts and medical files were developed in a multidisciplinary team (medical psychologists and oncologist) based on those of a previous RCT and were adjusted based on a pilot study.^28,29^

### Analysis

All audio recordings were transcribed verbatim into MAXQDA.^30^ All transcripts were independently content coded by 2 coders (L.F.v.d.W. and G.C.S.).^31^ Data were analyzed by means of content analyses.^32^ Moreover, we used a combination of an inductive and a deductive approach. Verbal expressions referring to information provision (including information about side effects and complications) and decision making (including the treatment options) were coded inductively; that is, the coding scheme was not prespecified but developed parallel to the coding of the transcript. After every few transcripts, ES joined in to triple code a transcript. Results of every transcript were jointly discussed by the coders to reach consensus on the initial coding scheme. Throughout the process, the coding scheme was further refined until after 10 transcripts only few new codes emerged from consensus discussions. The 9 previously analyzed transcripts were reviewed to make any complementary coding adjustments based on the final coding scheme

When critically reviewing and thematically categorizing the comprehensive coding scheme, it emerged that HCPs’ recommendations and preferences were regularly expressed in a rather implicit manner. This is in line with literature on implicit persuasion.^33,34^ Therefore, we continued our analyses more deductively, using the descriptions of ways of implicitly steering a choice as described in this literature as codes.

In addition, a deductive approach was followed by rating all consultations on the degree to which the HCP involved the patient in decision making using the OPTION-12.^35^ This quantitative measure was added to help gain a complete picture of the decision-making process and allow for comparison with other cancer settings. The OPTION-12 is a coding instrument to assess the extent that SDM is happening in a health care consultation, from the perspective of trained observers who use a recording of the actual consultation and the General OPTION-12 manual. Items are rated on a 5-point Likert-type scale (0 = *not observed* to 4 = *very high standard*), and the sum score is transformed to reflect a total out of 100. Two assessors independently rated the recorded consultations. The coding process consisted of training, calibration to achieve sufficient interrater reliability, and independent coding. Since intraclass correlation coefficients and kappa values were not considered sufficient for independent coding, all consultations were double coded and scores averaged or discussed until consensus was reached. Item 12 of the OPTION-12, “The clinician indicates the need to review the decision (or deferment),” was deemed not applicable to this specific setting, as the decision making for this case usually requires the patient to first consult multiple other HCPs before a final decision can be made. Therefore, the summed OPTION scores were calculated by using the mean score of item 1 to 11 and scaling it to a score between 0 and 100, following the guidelines.^36^

Findings are presented as frequency of occurrence and illustrated with qualitative descriptions.

## Results

Twenty SPAs of 20 different HCPs were included in this study, of which 11 were surgical and 9 radiation HCPs. Descriptive characteristics are shown in Table 1. The HCPs were on average 47.6 y old and were most often male (60%). The mean duration of the SPA consultations was 33.9 min (*s* = 10.3 min) and ranged from 21.1 to 57.5 min. The scores for realism and comparability to clinical practice for the SPAs were 7.8 and 7.0 (on a 1–10 Likert-type scale), respectively.

**Table 1:** Descriptive Characteristics of the Participating Health Care Providers and Standardized Patient Assessments for Surgical and Radiation HCPs Separately

	Total Sample	Surgical HCPs	Radiation HCPs
*n*	20	11	9
Age, y, $\bar{x}$ (*s*)	47.6 (7.4)	47.7	47.4
Gender, male, *n* (%)	12 (60)	9 (82)	3 (33)
Center employed at, academic (v. nonacademic), *n* (%)	12 (60)	6 (55)	6 (67)
Job position, *n*			
Medical specialist (senior staff)	18	9	9
Physician assistant	1	1	0
Nurse specialist	1	1	0
Duration of standardized patient assessment, min, $\bar{x}$ (*s*)	33.9 (10.3)	35.04 (12.9)	32.4 (6.1)

### Presenting the Treatment Options

#### What information about treatment options is provided

Between 1 and 4 treatment options were discussed per consultation, from a total of 5 different treatment options that were observed in total. Many HCPs presented 2 options (7/20). Observed treatment options are shown in Tables 2 and 3.

**Table 2:** Treatment Options Discussed by Health Care Providers Accompanied by the Number of Health Care Providers Discussing the Option

Treatment Option	Frequency (/Total)	Meaning
Neoadjuvant chemoradiotherapy+surgery (nCRT + surgery)	20 (/20)	A combination of chemotherapy and radiotherapy (5 wk), followed by surgery
Definitive chemoradiotherapy (dCRT)	15 (/20)	Combination of chemotherapy and radiotherapy (6 wk)
“Doing nothing” or “treatment aimed at symptom relief”	10 (/20)	Palliative care without anticancer therapies (best supportive care)
Neoadjuvant chemoradiotherapy followed by wait-and-see policy (nCRT + wait-and-see)	6 (/20)	A combination of chemotherapy and radiotherapy (5 wk), followed by scans and endoscopies to evaluate whether surgery should take place or not
Surgery only	3 (/20)	Surgery without (neo)adjuvant treatment

**Table 3:** Treatment Options Discussed per Health Care Provider (HCP),^a^ Subdivided for Hospitals (Center A–H) and HCP Specialty^b^

Treatment Option	Center A					Center B			Center C		Center D		Center E			Center F		Center G	Center H	
nCRT + surgery	S	S	S	R	R	S	S	R	S	R	R	R	S	S	S	R	R	R	S	S
dCRT	S	S	S	R		S	S	R	S	R	R	R			S		R		S	S
“Doing nothing”							S	R	S	R	R		S		S			R	S	S
nCRT + wait and see													S	S	S			R	S	S
Surgery only										R							R	R		

dCRT, definitive chemoradiotherapy.

a One HCP = 1 column.

b S = surgical HCP; R = radiation HCP.

HCPs from within each center as well as between centers varied in which (combination of) treatment options they discussed with the patient (see Table 3). All HCPs discussed nCRT followed by surgery as a treatment option, including 1 HCP mentioning only this 1 option. nCRT followed by a wait-and-see policy was discussed by 6 HCPs from 3 centers, all of which participated in a study investigating this latter approach.^37^ Aside from other curative-intent options (e.g., definitive chemoradiotherapy or surgery only), 10 of 20 HCPs also mentioned “doing nothing” or “treatment aimed at symptom relief,” rather than cure, as an option.

#### How information about treatment options is provided

Every HCP mentioned nCRT followed by surgery as the first option and informed on this option quite extensively. After doing so, most HCPs introduced the other possible treatment options halfway through the consultation or later and often used fewer words to explain these. By doing so, emphasis was put on nCRT followed by surgery throughout most of the consultations.

### Presenting a Choice

#### What information on treatment choice is presented

In most of the consultations (16/20), an agenda was set at the start of the consultation. The content of the agenda varied between HCPs; some HCPs mentioned the existence of multiple treatment options or possibilities (see quote 1); others merely announced that they were going to inform the patient about treatment or explicitly introduced their treatment proposition (quote 2). Hardly any HCP (3/20) first introduced the treatment options they were going to discuss, before starting to inform the patient on the first option.

Quote 1. Radiation oncologist: “There are **multiple treatment options**, which I would like to discuss with you, including the pros and cons, after which I think we need to look **together** at which treatment would be the best option for you.”
Quote 2. Surgeon: “We have also discussed your case with the entire team specialized in esophageal cancer, which includes the oncologist, radiation oncologist, gastroenterologist, us and several other specialists. Our **treatment proposition** is to start with radiotherapy combined with chemotherapy, about which the radiation oncologist and medical oncologist will further inform you.”

#### How treatment choice is discussed

Throughout the consultation, all HCP used at least 1 utterance suggesting a choice, whether explicitly or implicitly. Frequently, a choice was only very implicitly suggested, by using the words *advice* or *proposition*, implying the possibility for the patient to not accept the proposed (see quote 2). However, most HCPs also more explicitly mentioned the existence of a choice between multiple options, using for instance the words *choosing* or *option* (see quote 1).

### Informing about Pros and Cons of Treatment(s)

#### What information about pros and cons is provided

##### Side effects and complications

Quite some variation was observed in the side effects and complications HCPs discussed, ranging from 0 to 11 side effects and complications per consultation. A total of 28 different side effects and complications were observed throughout all consultations, which included multiple side effects that were observed for several different treatment modalities (see Tables 4 and 5). Almost all surgical HCPs discussed anastomotic leakage and pneumonia as possible complications of treatment. However, surgical HCPs varied in which other side effects and complications they discussed (see Table 4). In some consultations (3/11), the surgical HCP did not mention any specific side effects or complications but described a more general impact of treatment (see quote 3).

**Table 4:** Side Effects and Complications Communicated by Surgical Health Care Providers (1 HCP = 1 Column)^a^

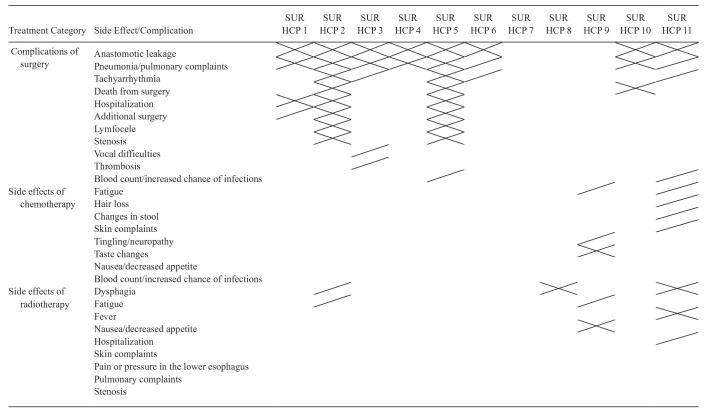

HCP, health care provider; SUR, surgical.

a No filling = not addressed, / = shortly addressed (i.e., in enumeration), X = more than shortly discussed (e.g., including expression of symptoms, supplementary medication, etc.).

**Table 5:** Side Effects and Complications Communicated by Radiation Health Care Providers (1 HCP = 1 Column)^a^

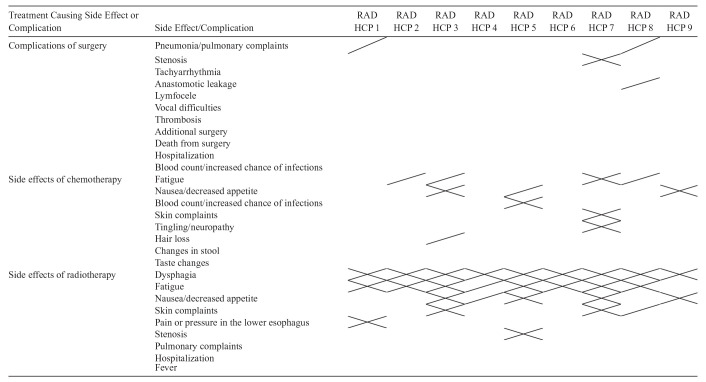

HCP, health care provider; RAD, radiation.

a No filling = = not addressed, / = shortly addressed (i.e., in enumeration), X = more than shortly discussed (e.g., including expression of symptoms, supplementary medication, etc.).

Quote 3. Surgeon: “And, well, the surgery, we will discuss that in a minute. It’s **not a minor surgery**, though it’s . . ., we will perform a keyhole surgery, but it’s still **major** surgery. It’s one of the **most invasive** types of surgery you could inflict on a patient if I’m being honest.”

Radiation oncologists generally discussed at least dysphagia and fatigue as possible side effects of radiotherapy (see Table 5). Regarding the additional side effects discussed, radiation oncologists again showed some variation. Most of the HCPs (14 out of 20 surgical and radiation HCPs) discussed some of the side effects of chemotherapy in addition to the side effect information of their own expertise.

##### Quality of life and recovery

Many HCPs (17/20) discussed the treatments’ possible effects on a patients’ quality of life or daily living. Some of these HCPs addressed a higher or lower level of general quality of life, specifically using the term *quality of life*, as being a pro or con of treatment options. Other HCPs discussed only the consequences that treatment (e.g., surgery) might have for patients’ daily lives (see quote 4). Also, in many consultations (14/20), the physical recovery from surgery or chemoradiotherapy was discussed by the HCP.

Quote 4. Radiation oncologist: “Well, yeah, the changes after the esophageal surgery, those are more serious. They will thoroughly change **your eating habits and lifestyle**, especially for the first 6 to 12 months after surgery.”

### How Information about Pros and Cons Is Provided

#### Comparing pros and cons

While almost none of the HCPs labeled the implications of treatment as being either a pro or a con of treatment, they regularly used another form of guiding the patients through the tradeoff of treatment outcomes. Many HCPs, especially those discussing dCRT as an option, compared the pros and cons of nCRT followed by surgery to those of other treatment options (e.g., dCRT; see quote 5). HCPs, however, also frequently compared the pros and cons within 1 treatment option instead of between multiple options (see quote 6).

Quote 5. Radiation oncologist: “And there is an alternative that offers lower chances of cure, **but** it is less invasive.”
Quote 6. Surgeon: “Well, all in all. It’s a major treatment. A long treatment plan with major surgery at the end, erm, **but** this whole track including pretreatment and surgery will be your best option.”

Within the comparisons, survival was generally discussed as being the pro of nCRT followed by surgery and the con of other treatment options. However, HCPs used several different implications as pros of other treatment options to put against survival, such as quality of life (chance of) complications, (period of) recovery, or in more broad terms, the (smaller) “investment” associated with treatment.

In many cases (13/20), HCPs additionally concluded their consultation with a comparative summary containing the pros and cons of the discussed treatment options (see quote 7).

Quote 7. Radiation oncologist: “That’s an alternative which offers a lower chance of cure. Whereas the chance of cure with surgery is 50%. So having 50% of patients survive after 5 years, is definitely something we take into consideration. And if we look at just radiation and chemotherapy that’s about half.”

### Communicating Recommendations

About half of the HCPs gave explicit advice to choose nCRT followed by surgery, for instance by using the words *(treatment) proposition*, *advice*, or *(treatment) plan*.

Some HCPs presented their advice or recommendations as being one of their own (using *I* or *mine*), whereas many others stated the advice as being one of the team of treating HCPs (using *we* or *our*). In many consultations, the explicit advice was given already at the start of the consultation.

In addition, more implicit ways of HCPs declaring their personal preference for nCRT followed by surgery were observed, which are presented below

#### Authorized decision

As mentioned, HCPs often presented their preference as one of the team of treating HCPs or “experts,” suggesting authorization of the defined “we” (see quote 2). In some other cases, HCPs presented their preferred treatment as an authorized proposition based on “the guidelines” (see quote 8).

Quote 8. Surgical nurse specialist: “**According to guidelines**, the **most ideal** treatment is: radiation therapy combined with chemotherapy for 5 weeks. Followed by surgery.”

#### Value judgment

Another frequently used manner of marking their personal preference was HCPs using labels that suggest a value judgment. When introducing their preferred option, HCPs, for instance, called this option “the best treatment” or “the treatment with the best chances.” Other HCPs used labels such as “the most appropriate” or “the most ideal” (see quote 8). Related to these value judgments, HCPs frequently used labels such as “standard treatment” or “the gold standard” (see quote 9).

Quote 9. Radiation oncologist: “It means, basically, also, given your age and, frankly, your fitness, that **you are eligible for standard treatment**, so to speak. And this is something the gastroenterologist will have mentioned, but standard treatment would be surgery before radiation and chemotherapy.”

Labels suggesting a value judgment were also used by HCPs when presenting the other, less preferred, treatment options. About half of the HCPs used the word *alternative* when introducing these treatments, suggesting a slight inequality between them. Other, more pronounced labels observed were “not an equal alternative,”“second best,” or even “hypothetical.”

#### Eligible for treatment

When HCPs presented their preferred treatment, some linked this treatment to the patient’s chances of cure based on clinical characteristics, for instance, by using phrases like “you are eligible for curative treatment” (see quote 9), by which they make clear that curative treatment is an option for this patient. HCPs hereby seem to suggest that specific patients are eligible for certain treatment options while leaving out how well certain treatment options fit to the preferences of a specific patient. More surgical than radiation HCPs (4/5) seemed to use this practice.

#### Offering you the best possible outcomes

Another way HCPs might link their preferred treatment to the chances of a cure is by framing the treatment as an option they can personally offer to the patient, as seen in quote 10. This approach could suggest a personal preference of the HCP for that specific treatment option.

Quote 10. Surgeon: “Alright, well, the fact that it hasn’t metastasized in other organs, is indeed, as you mention, good news. Because that means we can essentially **offer you** a treatment plan with the highest chance of cure.”

### Involving Patients in Decision Making

The first moment at which HCPs explicitly involved patients in making the decision appeared to be most often halfway or to the end of the consultation, mostly when HCPs were exploring the patient’s preferences or working toward a decision. HCPs rarely explicitly mentioned the patients’ role in decision making during the consultation (such as in quote 1). The patients’ preferred level of involvement in the decision making was discussed in none of the consultations.

However, when working toward a decision near the end of the consultation, some HCPs did ask patients how or when they would prefer to decide on treatment. These HCPs, for instance, introduced the patients’ next consultation with one of the other HCPs in the trajectory or suggested patients to consult their family, general practitioner, or a geriatrician for deliberation. Often, these HCPs also suggested patients plan an extra consultation with them afterward, to finalize their decision. In other consultations, HCPs merely asked for patients’ consent to their proposed treatment. In these, a frequently stated proposition (6/20 consultations) was for patients to already start nCRT and optionally postpone the decision on which treatment would follow, being surgery, wait-and-see policy, or definitive CRT.

The mean OPTION12 score across all consultations was 40.11 (*s* = 12.42), ranging from 18.2 to 67.0 (see Table 6).

**Table 6:** Health Care Providers’ Mean OPTION-12 Total Scores and Individual Item Scores

	$\bar{x}$ (*s*)
OPTION-12 total score (0–100)	
Total	40.11 (12.42)
Surgical HCPs	39.15 (9.98)
Radiation HCPs	41.28 (15.47)
OPTION-12 item score (0–4)	
Item 1. The clinician draws attention to an identified problem as one that requires a decision-making process	2.20 (1.07)
Item 2. The clinician states that there is more than one way to deal with the identified problem (“equipoise”)	2.30 (1.07)
Item 3. The clinician assesses the patient’s preferred approach to receiving information to assist decision making	0.25 (0.44)
Item 4. The clinician lists “options,” which can include the choice of “no action”	1.55 (0.92)
Item 5. The clinician explains the pros and cons of options to the patient	2.48 (0.97)
Item 6. The clinician explores the patient’s expectations (or ideas) about how the problem(s) are to be managed	1.35 (1.31)
Item 7. The clinician explores the patient’s concerns (fears) about how problem(s) are to be managed	2.08 (0.41)
Item 8. The clinician checks that the patient has understood the information	1.80 (0.82)
Item 9. The clinician offers the patient explicit opportunities to ask questions during the decision-making process	2.18 (0.85)
Item 10. The clinician elicits the patient’s preferred level of involvement in decision making	0.33 (0.52)
Item 11. The clinician indicates the need for a decision-making (or deferring) stage^a^	1.15 (1.01)

a Item 12 of the OPTION-12—“The clinician indicates the need to review the decision (or deferment)”—was deemed not applicable to this specific setting, as the decision making for this case usually requires the patient to first consult multiple other HCPs before a final decision can be made. See the “Analysis” section (“Methods” section).

## Discussion

The present study shows extensive practice variation in how HCPs involved standardized simulation patients with localized esophageal cancer in decision making. In some consultations, HCPs presented a choice between options and involved patients in decision making, others merely communicated a treatment recommendation, and some combined the two. This range suggests a lack of consensus between clinicians on how to approach decision making for these patients. Variation in the decision-making process throughout consultations could normally be a sign of the HCP tailoring to the specific preferences of a certain patient. However, the fact that in this study the patient was standardized suggests that HCPs might not very well be able to distinguish or attune to a patient’s needs or preferences, which is in line with literature on person-centered decision making.^38,39^

Second, large variation was observed in the number and type of treatment options and pros and cons of these options that were presented to patients. Interestingly, a similarly large variation of addressed benefits and harms was found in an analysis of consultations on preoperative radiotherapy for rectal cancer.^40^ HCPs should deliberate on the desirability of such large variation in both pros and cons and options, as this means that it makes a difference which HCP a patient consults. In any case, HCPs should at least provide a minimal agreed upon amount of information, especially about the pros and cons, as it is necessary for informed consent and SDM alike that patients receive sufficient information on the possible outcomes of treatment options.^41^

Both the lack of consensus on which treatment options and outcomes to provide to patients and on how to involve patients in the choice between these options thus call for some form of mutual alignment between HCPs. One opportunity for daily clinical practice might be to grant a more central role to the multidisciplinary meetings by using them to collectively decide which treatment options to discuss with a specific patient, instead of deciding on the teams’ treatment proposition. The role of patient preference in the treatment choice could potentially also be subject to multidisciplinary discussion. Regarding the side effects and complications of treatment, HCPs of each discipline might benefit from establishing a core set of information that patients should receive when considering each treatment, using, for instance, a Delphi approach and possibly also including patient perspectives.^40^ Protocols as well as patient information materials, such as flyers or websites, could subsequently be adjusted to include these.

Third, aside from explicit forms of presenting a choice or giving advice, we observed many implicit forms of doing so. Regarding the presentation of a choice, the order in which HCPs addressed the different options and the amount of information they gave about each option may—wittingly or unwittingly—create a focus on one of the options. Furthermore, HCPs should be aware that the information that they present at the start of the consultation (i.e., introducing a treatment proposition or plan rather than stating that multiple options should be considered) could affect the extent to which patients listen and participate actively during the rest of the consultation and consequently affect the treatment decision.^19^ Another notable finding was the way in which HCPs framed nCRT followed by surgery (e.g., using value-judging labels when introducing this treatment option, such as being the “gold standard” or offering them “the best possible outcomes”) but also emphasizing the fact that patients “are eligible” for this treatment. With all of the above behaviors, HCPs might consciously or unconsciously steer patients toward choosing a specific option.^33^ For instance, by stating patients “qualify for surgery,” instead of presenting surgery as an option that might fit them, patients might feel like being one of the “winners” and subsequently not think they have a real choice.^33,42^ HCPs’ desire to steer their patient toward the option that they believe is in their best interest could be considered commendable.^33^ When it is the case, however, that the persuasion behaviors occur unconsciously, HCPs might find themselves in a situation in which their behavior does not match their actual intention to present patients with a true choice. Consequently, patients might think they do not have a choice or even be afraid to not accept the HCP’s advice.^43^ To change these potentially unconscious and habit-like behaviors, HCPs could benefit from communication training showing the possible effects on patients and offering alternatives.

When quantifying the amount of SDM used by HCPs, a fairly higher mean OPTION score ( $\bar{x}$ = 40) emerges compared with the mean OPTION score of 23 as reported in a review of the literature among several diseases from 2003 to 2015.^44^ Our data may reflect the increasing acceptance and implementation of SDM in clinical practice in recent years.^45^ More recent studies, such as the CHOICE (2015–2016) and the SYMPHONY (2020–2021) trial, involving cases in the palliative esophagogastric setting, report mean OPTION scores of 34.6 (*s* = 11.6) and 43.13 (*s* = 12,28), respectively.^45^ Interestingly, OPTION scores in the current curative setting do not differ much from these palliative SPA scores, while generally, SDM seems to be more widely accepted in the palliative oncologic setting in contrast to the curative setting. This suggests less difference than anticipated and may give occasion for further research on the level of patient engagement in decision making in this and other oncologic settings. Nonetheless, it may be argued that SDM is suboptimal, as the average OPTION score was still far from the maximum score of 100, suggesting room for improvement. Of note, the lowest scores were on items that are rather idealistic (i.e., assessing patients’ preferences for different information formats).^46^ The highest scores regarded information provision skills and the lowest communication about patients’ values and emotions. Whereas the first set of skills is mostly— although not exclusively—about sending messages, the latter requires a more receptive and exploring attitude and may be more difficult for HCP.^45^ In addition, as mentioned, we found HCP to explicitly and implicitly present information in such a way that it could have impeded patients’ perception of having a choice. This element of decision-making conversation is not incorporated in the OPTION.

A strength of the currently used, standardized design is that it enabled us to focus on HCPs’ behavior and variation thereof, largely ruling out the effect of patient variation. The design was further strengthened by including HCPs from different professions and from many different hospitals, both academic and nonacademic. However, the design prevented us from looking into the way that patient preferences and patient and HCP interaction might affect decision making, aspects that should not be neglected when describing real-life practice. Other limitations of the current design include matters of resemblance of the simulated consultations to a real-life situation, that is, the largely online setting of the simulated consultations due to COVID-19 and the fact that the cases represent a first consultation about treatment options, while some HCPs usually consult the patient after 1 or multiple consultations have been performed by their colleagues from the team of treating HCPs. In addition, the use of SPA could have influenced the perceived realism and comparability to clinical practice, thereby affecting the HCPs’ performance, although both of these parameters were scored as acceptable (>7) by the HCPs themselves.

HCPs were unaware of our specific interest in assessing the level of SDM, yet they were aware that we investigated the provision of information. Considering the simulated nature of the study, a Hawthorne effect of HCPs’ intentionally increasing their information provision cannot be ruled out.^47^ We could not put the performance of the HCPs in perspective because we had no baseline assessments of their proficiency. Yet, there is little evidence for an effect on behavior when HCPs are aware of being video recorded.^48^ Lastly, the frequencies and OPTION scores as presented in this article should be interpreted cautiously in view of the small sample size and simulated setting.

To assess decision making in real-life clinical practice, the multidisciplinary nature of this setting should certainly not be neglected. For instance, the role of the medical oncologist in the decision-making process should also be examined. Future research investigating the interactions between patients and all of the involved HCPs over the different consultations and the division of roles between different HCPs in turn consulting the same patient could provide valuable additional insights into the decision-making process in clinical practice.

## Conclusions

The extensive practice variation within HCPs consulting the same (simulated) patient demonstrates a need for further alignment between HCPs in the curative esophageal setting. Alignment is needed in 1) how to involve the patient in decision making, 2) which treatment options HCPs should discuss, and 3) which pros and cons of these options HCPs should minimally inform the patient about. HCPs must furthermore be aware of the possible effects of certain, conscious or unconscious, steering behaviors on a patient’s choice. Training in SDM and communication skills is needed for HCPs to attune their intentions regarding decision making to their behavior.

## Supplemental Material

sj-docx-1-mpp-10.1177_23814683251349473 – Supplemental material for Decision Making about Localized Esophageal Cancer Treatment: An Observational Study on Variation in Clinicians’ Communication Behavior Supplemental material, sj-docx-1-mpp-10.1177_23814683251349473 for Decision Making about Localized Esophageal Cancer Treatment: An Observational Study on Variation in Clinicians’ Communication Behavior by L. F. van de Water, G. C. Scholten, I. Henselmans, J. Heisterkamp, P. M. Jeene, F. F. B. M. Heesakkers, K. J. Neelis, B. R. Klarenbeek, M. I. van Berge Henegouwen, J. W. van den Berg, J. Buijsen, E. D. Geijsen, H. W. M. van Laarhoven and E. M. A. Smets in MDM Policy & Practice
