# Supplementary material for: Decision Making about Localized Esophageal Cancer Treatment: An Observational Study on Variation in Clinicians’ Communication Behavior
Source: MDM Policy Pract. 2025 Jun 30;10(1):23814683251349473. doi: 10.1177/23814683251349473 (PMC12214366; doi:10.1177/23814683251349473)
Supplement: sj-docx-1-mpp-10.1177_23814683251349473 – Supplemental material for Decision Making about Localized Esophageal Cancer Treatment: An Observational Study on Variation in Clinicians’ Communication Behavior [file sj-docx-1-mpp-10.1177_23814683251349473.docx]

Appendices

Appendix 1. Standardized Patient Assessment (SPA) cases

|  | **Curative case 1** | **Curative case 2** |
| --- | --- | --- |
| **Sex** | Male | Male |
| **Age** | 76 | 74 |
| **Occupation** | Retired accountant | Retired soil engineer |
| **Type of cancer** | Localized oesophageal | Localized oesophageal |
| **Indicated treatment** | Curative treatment; fit for surgery | Curative treatment; fit for surgery |
| **Tumor** | Squamous cell carcinoma | Squamous cell carcinoma |
| **TNM** | T3N1M0 | T3N1M0 |
| **WHO** | WHO-1 | WHO-1 |
| **Comorbidities** | Hypertension; heart attack (10 years ago) | Hypertension; type 2 diabetes; arthrosis |
